# Involving Knowledge Users in Health Services Research: Collective Reflections and Learning From a National Evaluation of Recurrent Miscarriage Services

**DOI:** 10.1111/hex.70125

**Published:** 2024-12-16

**Authors:** Marita Hennessy, Rebecca Dennehy, Hannah O'Leary, Keelin O'Donoghue, RE:CURRENT Research Advisory Group, Una Cahill, Ríona Cotter, Mairie Cregan, Carrie Dillon, Linda Drummond, Angela Dunne, Minna Geisler, Trish Horgan, Azy Khalid, Con Lucey, Mary McAuliffe, Moya McMenamin, Yvonne O'Brien, Orla O'Connell, Anne O'Flynn, Aideen Quigley, Margaret Quigley, Rachel Rice, Noirin Russell, Jennifer Uí Dhubhgain, Anna Maria Verling, Jill Whelan

**Affiliations:** ^1^ Pregnancy Loss Research Group, Department of Obstetrics and Gynaecology University College Cork Cork Ireland; ^2^ INFANT Research Centre University College Cork Cork Ireland

**Keywords:** knowledge translation, knowledge user engagement, miscarriage, participatory approaches, patient and public involvement

## Abstract

**Introduction:**

Involving knowledge users in research can facilitate the translation of evidence into policy and practice. How to best involve and support various types of knowledge users, including patient and public involvement contributors, in research is an identified knowledge gap. We conducted a national evaluation of recurrent miscarriage care supported by a Research Advisory Group (convened in March 2020) comprising a range of knowledge users, including parent advocates and people involved in the management/provision of services. The Group met virtually nine times, and actively collaborated beyond this on various research activities across the project. In this paper, we share insights from our collective evaluation of these involvement efforts.

**Methods:**

We drew on records kept over the timespan of the project to describe involvement activities and experiences. Advisory Group members participated in an electronic survey to assess their involvement experiences at two time points (February 2021 and May 2022); we analysed the results descriptively. In May 2022, we hosted a virtual World Café, comprising the Research Team and Advisory Group, to explore what worked well and what could have been improved regarding involvement activities within the project; we analysed this data thematically.

**Results:**

Responses to both rounds of the survey were positive, with people reporting: their ability to discuss research issues, contribute to the research, express their own views; feeling valued as a partner; that they could bring their own ideas and values to the research; perceived potential to gain status, expertise, or credibility because of their involvement. Themes constructed from the Word Café discussions highlighted that structural and relational spaces shaped the accessibility and experience of involvement.

**Conclusion:**

Members reported a positive and rewarding experience with a visible impact on the research process but highlighted issues with the feasibility and scope of the research protocol and challenges to autonomous involvement in aspects reliant on clinical expertise. Our analysis reinforces that the relational nature of involvement takes precedence over instrumental aspects or techniques. Realistic study protocols that allow time and space for the evolving nature of research with knowledge users, and institutional and financial support to facilitate meaningful involvement, are needed.

**Patient or Public Contribution:**

People with lived experience of recurrent miscarriage/pregnancy loss were involved in this evaluation—as members of the RE:CURRENT Research Advisory Group, contributing to the methodology, evaluation activities, interpretation and reporting of findings and insights.

## 1 Introduction

Engaging people who can benefit from research (‘knowledge users’) in research is advocated as an approach to enhance research relevance, usefulness and impact, thereby facilitating the translation of research findings into policy and practice [1, 2, 3, 4]. Various terms are used to describe such research partnerships, including community‐based participatory research, participatory action research, patient and public involvement (PPI) and integrated knowledge translation or ‘research coproduction’ [3, 5]; ‘engaged research’ or ‘research engagement’ are also used [6]. There is a ‘crowded landscape’ of PPI‐related terminology within and across countries also, with terms such as consumer or service user involvement or engagement, co‐design and co‐production used, often interchangeably [7].

Collaborative research approaches to generating and translating knowledge are more similar than different to each other; they require time and financial investment, and share core values and principles such as reciprocity, trust, respect, shared decision‐making and active participation [8]. However, there is a lack of evidence that the use of ‘co’ approaches (such as co‐production and co‐design) leads to improved health outcomes [9]. Furthermore, while there is now much guidance to support PPI in research [10], evidence to support best practices for integrated knowledge translation is lacking [4], and the process of engaging a broad range of knowledge users is an identified knowledge gap [11]. Reviews have noted a lack of reporting on how and/or when knowledge users are engaged in various stages of research [3]. Tailored approaches, negotiated and agreed at each stage with the people involved, are needed to support public involvement [12]; the quality of involvement takes precedence over the methods [8, 12, 13]. To integrate involvement in research, reflexivity and supporting relationships are required, whereby space is made for critical discussion and changing course where necessary [13].

Despite an increased emphasis on knowledge user involvement in research and practice in recent years, evaluation is limited [14]. There is debate about where the focus of evaluation efforts should lie, related to underlying reasons for undertaking co‐produced research, particularly regarding PPI, and whether impact evaluation is appropriate [15, 16]. Furthermore, different approaches to evaluating PPI can be taken based on the values and goals underpinning it—whether it is a technocratic process led by researchers to improve research quality and/or a democratic one which sees involvement as a right and seeks to challenge power structures [17, 18]. As such, the approach taken can distort how PPI is conceptualised and practised, with the potential to increase performativity and downplay negative impacts [19]. Context, process, effects and impacts are key factors in the evaluation of knowledge translation strategies [20]. While advocated, there is limited literature describing the optimal processes for research coproduction with various types of knowledge users and limited evidence supporting the impacts of partnership working [3, 21].

Regarding the evaluation of public involvement in health research specifically, a wide variety of methods are used, across a range of aspects including empowerment, impact, respect, support and value [22]. Increasingly researchers and PPI contributors are sharing experiences of PPI [13, 23], with the aim of improving practice. However, while the benefits of research partnerships have been noted, so too have negative impacts, such as feelings of tokenism, disempowerment and being over‐burdened [3]. Researchers may choreograph PPI ‘to respond to and impress the funder's gaze' which can result in inauthentic or insincere involvement, constrained by time and resources [24]. All aspects of PPI—both positive and negative—need to be reflected on and discussed on an ongoing basis [25].

In this article, we aim to share our experiences of embedding knowledge user involvement in a national evaluation of recurrent miscarriage services. This formed part of our strategy to enhance the uptake of knowledge generated into policy and practice. We were conscious that combining different types of expertise within a project could be difficult in practice, with the potential for certain issues, people and knowledge to be prioritised at the expense of others [26]. This said, we began our project from the position that involving knowledge users from the outset would add value; a consideration that we engaged reflexively around. The Research Group (Pregnancy Loss Research Group) has a history of PPI, with parent advocates included within the membership, monthly meetings, and research activities [27]. Similar to Gibson et al., we focused our evaluation on assessing and improving the quality of interactions with and between knowledge users to make a difference in how we understand, and affect change in, recurrent miscarriage services and supports [28].

Our primary aim in embedding an evaluation of knowledge user involvement within the project was to inform our own activities and shared sense‐making, identifying what works in building and sustaining involvement and to enable us to change course if needed, to support meaningful involvement. Longitudinal evaluation of involvement activity facilitates this [29]. Our secondary aim was to share learnings and provide insights into how individuals, groups, funding agencies and institutions can better support and integrate knowledge user involvement in research [30]; the focus of the current paper. We describe the processes of involving knowledge users within our project, and our collective views and experiences of involvement based on our evaluation.

## 2 Materials and Methods

We adopted an integrated knowledge translation approach to research partnership/co‐production as researchers working with knowledge users on the RE:CURRENT (Recurrent miscarriage: Evaluating current services) project; a national evaluation encompassing six work packages which are outlined later in Table 1 [31]. Knowledge translation activities were underpinned by the Knowledge to Action Framework [32]. With and/or through the Advisory Group we adopted several knowledge translation strategies outlined in the ERIC taxonomy [33].

**Table 1:** Project activities and knowledge user involvement.

WP	Project activities	Knowledge user involvement
1	Identification, synthesis and appraisal of clinical practice guidelines for recurrent miscarriage care	Involved in the development of a protocol for the systematic review and the interpretation of findings Authorship on both published papers—protocol and review (R.R.)
2	Evaluation of service provision in the Republic of Ireland against guideline‐based key performance indicators (KPIs) for recurrent miscarriage care	Agreed and participated in six‐stage process to develop guideline‐based KPIs, including two‐round modified e‐Delphi survey, four virtual consensus meetings, a final survey and virtual meeting Authorship on published papers—development of KPIs (R.R.); service evaluation (R.C., O.O.C.)
3	National care experience survey	Nine meetings of the Advisory Group; involvement in other work packages highlighted above. Participated in evaluation of quality of involvement (two surveys, World Café) Participated in the development of national clinical guidelines for recurrent miscarriage Contributed to research papers, videos, infographics, media articles, blogs, project updates
4	Qualitative interview study of knowledge user views and experiences of recurrent miscarriage services	Nine meetings of the Advisory Group; involvement in other work packages highlighted above. Participated in evaluation of quality of involvement (two surveys, World Café) Participated in the development of national clinical guidelines for recurrent miscarriage Contributed to research papers, videos, infographics, media articles, blogs, project updates
5	Health economic analysis: (1) costs associated with the implementation of a best practice model of care; (2) impacts of receiving care	Nine meetings of the Advisory Group; involvement in other work packages highlighted above. Participated in evaluation of quality of involvement (two surveys, World Café) Participated in the development of national clinical guidelines for recurrent miscarriage Contributed to research papers, videos, infographics, media articles, blogs, project updates
6	Integrated knowledge translation	Nine meetings of the Advisory Group; involvement in other work packages highlighted above. Participated in evaluation of the quality of involvement (two surveys, World Café) Participated in the development of national clinical guidelines for recurrent miscarriage Contributed to research papers, videos, infographics, media articles, blogs, project updates

*Note:* R.R., J.U.D., C.L.—parent advocates; R.C., O.O.C.—health professionals.

Abbreviation: WP = work package.

### 2.1 Overview of the Evaluation Design

**Figure 1:**
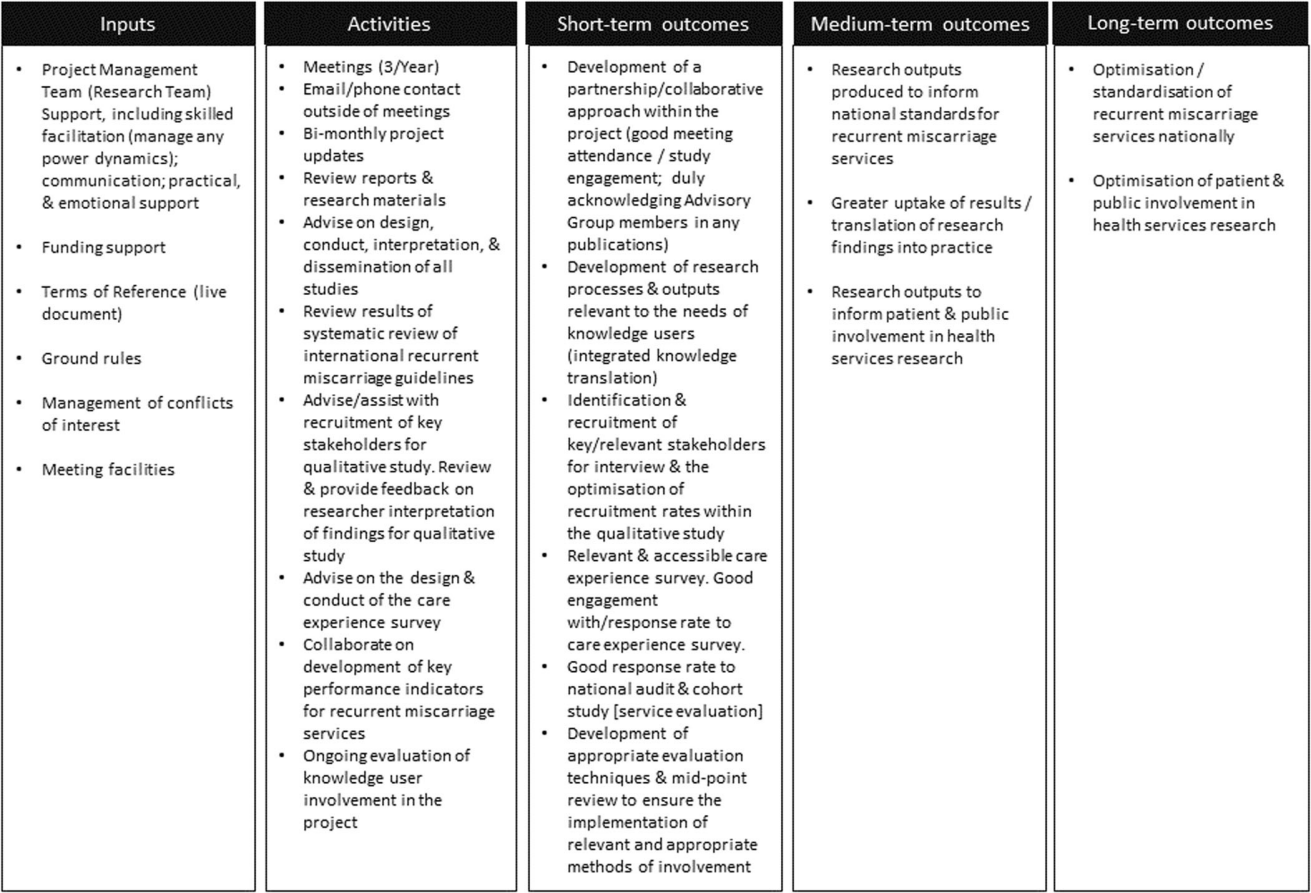
RE:CURRENT knowledge‐user involvement logic model.

At the outset of the Project, the research team (R.D., M.H., K.O.D.), in conjunction with the Advisory Group, developed a logic model to articulate how we anticipated knowledge‐user involvement in the RE:CURRENT project to work (Figure 1). An initial outline was drafted by the research team—drawing on relevant research—and was discussed and refined at the second Advisory Group meeting.

We adopted a mixed‐methods participatory approach to evaluate involvement activity. Given our aim to understand how we could better support and integrate knowledge user involvement in our project, our evaluation focuses on inputs, activities and some of the short‐term outcomes outlined in the model. Longer evaluation timeframes would be needed to explore the medium‐ to longer‐term outcomes. Evaluation design and methods were discussed and agreed by the Advisory Group in advance. This was important for meaningful involvement as PPI contributors and other knowledge users can have different views and we were committed to everyone being involved in discussions and decisions from the outset [16].

### 2.2 Ethical Considerations and Reflexivity

While this was an involvement activity (and thus did not require ethical approval [34]), we obtained ethical approval from the Clinical Research Ethics Committee of the Cork Teaching Hospitals (CREC) [Ref: ECM 4 (gg) 10/11/2020]. Beyond procedural ethics, as a research team, we were cognisant of attending to ‘everyday ethics’ [35, 36] and our ‘ethic of responsibility’ [13, 30] and care [37] in both the evaluation process and our ongoing interactions with the Advisory Group. We engaged in reflexive research practice throughout, as well as continuous dialogue regarding moral considerations, power and relational dynamics and the creation of opportunities for involvement.

We were also mindful of the unintended negative consequences of knowledge translation and engaged knowledge users and researchers in discussions to anticipate, mitigate or manage these [38]. This included briefing members on what would be involved in Group membership, having defined terms of reference and engaging in reflexivity. We recognised the potential for emotional harm or retraumatisation [39] of researchers and knowledge users, who may become distressed during the project, and/or engage in emotional labour [38]. We were conscious that building and sustaining relational and emotional inclusivity supports knowledge coproduction, and are negotiated through everyday interactions and practices [40].

### 2.3 Evaluation Methods

Various approaches to evaluating knowledge user engagement exist, including qualitative approaches, surveys and engagement logs [41]. We used mixed methods to inform our evaluation; these included a mid‐ and end‐point involvement quality survey, and World Café (Table 2). We also collated project records including meeting minutes, researchers' records of member input and actions taken, researchers' diaries and project outputs. This mixed methods evaluation was concurrent in design, with data from each phase collected and analysed separately. We compared findings across the phases, through the generation of a matrix and key themes, to inform our discussion.

**Table 2:** Overview of the evaluation methodology.

Phase	Evaluation method	Overview	Time period
1	Project records	The research team collated project records, e.g., meeting attendance logs and minutes, researchers' records of member input, details of decision‐making and actions taken, researcher diaries (M.H., R.D.), project outputs Aim: To chart inputs, activities and some of the short‐term outcomes of the project	Throughout the project: January 2020 to May 2022 (when the active engagement of the Advisory Group as a whole ceased)
2	Involvement quality survey	Advisory Group members completed a short questionnaire about the quality of their involvement experience Aim: To explore the quality of involvement experiences (short‐term outcomes) and identify areas (inputs, activities) to enhance or sustain	Mid‐point: February 2021 End‐point/Project completion: May 2022
3	World Café	Final (virtual) group meeting/activity incorporating the principles of World Café methodology. In small groups, Advisory Group members and research team members discussed involvement activities within the Project in line with the Logic Model and what worked well, what could have been better, and recommendations for future involvement efforts Aim: To explore the quality of involvement experiences (short‐term outcomes) and identify areas (inputs, activities) to enhance or sustain when involving knowledge users in research	May 2022

#### 2.3.1 Involvement Quality Survey

Many tools exist for evaluating health research partnership outcomes and impacts; these are mainly focused on outcomes, then process, and impact to a lesser extent [14]. A range of methods have also been used to evaluate PPI/community engagement in research, including a combination of qualitative methods such as interviews, focus groups, questionnaires and observations, with limited quantitative assessment [42]. More evaluation tools are now available to support PPI [43, 44, 45]; however issues with rigour and lack of PPI in their design and reporting have been noted [44]. We reviewed several tools, including those in Boivin and colleagues’ systematic review [44]. We decided to adapt the Quality Involvement Questionnaire to examine the nature and quality of knowledge user involvement in the project [46] as it was underpinned by theories of power and empowerment, in line with our values.

The Quality Involvement Questionnaire considers that quality involvement in health research encompasses interactions between personal factors and the research context. There are 31 questions across two categories, each with several domains (Table 3). While this questionnaire was developed to evaluate PPI in research, we felt it appropriate to examine the involvement of a broader range of knowledge users. On review by the Advisory Group during the evaluation planning phase, we excluded five items from the survey to keep it brief and directly relevant to our project.

**Table 3:** Items from the quality involvement survey included and excluded from our adapted survey.

Category	Domain (no. of items)	Item	Included in our survey
Personal factors	Ability (7)	Access research resources (e.g., money, facilities, information)	No
		Achieve your own goals through the research	Yes
		Make a contribution to the research	Yes
		Make decisions about how to do the research	Yes
		Express your views about research topics	Yes
		Discuss research issues	Yes
		Take on new research challenges	Yes
	Potential (5)	Choose the type of role you play in the research	Yes
		Bring your own ideas and values to the research	Yes
		Work in ways that suit you	Yes
		Gain status, expertise or credibility because of your involvement	Yes
		Identify and organise your research ideas and priorities	No
	Sense of being (5)	Valued as a partner (not controlled)	Yes
		Enabled (rather than constrained)	Yes
		Empowered (rather than exploited)	Yes
		Consenting (happy to be involved) not coerced (unhappy about it)	Yes
		It is accepted that different people have different responsibilities and decisions to make about the research	Yes
Research contexts	Research relationships (6)	The researchers have the right reasons for wanting to work with you	Yes
		There is sufficient funding to make involvement work	Yes
		You have enough information about involvement	Yes
		The way the researchers work with you is supportive	Yes
		The way the researchers communicate with you is supportive	Yes
		The types of goals that the researchers want are what you want	Yes
	Ways of doing research (4)	There is a clear role in the research for you	Yes
		The skills/experience needed for the role are clear to you	Yes
		The responsibilities for the role are clear to you	Yes
		You are aware of the legal and ethical ‘rules’ for doing research (e.g. confidentiality)	Yes
	Research structures (4)	Not just part of a project, it is valued as part of the work of the organisation	No
		Supported by research ethics and governance systems	No
		Helped because of research structures (networks, links with other studies, etc.)	No
		Noticed and recorded as part of the work of the organisation	Yes

*Note:* Participants are asked to rate their responses to each question from 0 to 4, where 0 is ‘not at all', 1 is low and 4 is high.

At the mid‐point of the Project—approximately 12 months (04–22 February 2021)—we invited the Advisory Group to complete the 27‐item electronic survey (hosted on QuestionPro) about the quality of their involvement experience. Following informed electronic consent, participants were asked to describe their role in the group (i.e., knowledge user type), and then answer questions relating to personal factors and research contexts. Results from Survey 1 were used to inform improvements for year two of the Project; they were discussed at an Advisory Group meeting (28 April 2021), and members were invited to give feedback, including on areas identified for improvement. The survey was repeated at the end of the project—approximately 24 months (10–31 May 2022)—to determine involvement quality in year two. We analysed the survey data descriptively in Microsoft Excel given the small number of participants and this analysis was checked by another member of the team. We calculated a mean score and range for each question and an overall mean individual score for the questionnaire (out of a potential total of 104), examining differences over time.

#### 2.3.2 Virtual World Café

At the final meeting of the Advisory Group, we used the World Café methodology to explore the involvement experiences of the Advisory Group and the research team (three postdoctoral researchers and the Principal Investigator), adapted for the virtual setting due to ongoing public health restrictions surrounding the COVID‐19 pandemic. World Café is an informal, flexible and effective format for hosting large group discussions and has been applied in many contexts [47]. The approach allows participants to share their viewpoints while considering alternative views and, in doing so, identify avenues for action.

Advisory Group and research team members were organised into four groups—Research Team, Health Professionals, Governance and Management and Parent Advocates. We did not mix knowledge users within groups as we felt that this may influence power dynamics and discussions. A note‐taker observed and took notes on interactions and key discussion points within each group. Three 10‐min rounds of discussion were facilitated, and participants were encouraged to record their ideas/points of discussion on a shared Padlet (https://padlet.com/). Participants were asked to consider which elements worked well or could have been better and make recommendations for future involvement efforts (see Supporting Information S1: File 1). At the end, all groups were invited to share insights from their discussions, along with Padlet data (an additional 30 min), with common themes identified and discussed by the overall Group.

The World Café was by facilitated by an experienced facilitator (D.D.) who had previously facilitated/chaired Advisory Group meetings, including consensus meetings regarding the development of key performance indicators (KPIs) [48]. Thus, the facilitator was known to all, and familiar with activities of the Group and Project. Additional details on the World Café are available in Supporting Information S1: File 2.

The main discussion at the World Café was audio‐recorded and transcribed through Microsoft Teams. This transcript was checked for accuracy, pseudo‐anonymised and imported to NVivo 12 [49], along with Padlet data. Reflexive thematic analysis, an iterative process of data familiarisation, data coding, theme development and revision, refinement and write up, was undertaken to explore patterns in the data [50]. It was led by RD, who met regularly with MH and KOD to discuss the analysis. Preliminary themes and sub‐themes were presented to the Advisory Group for review, discussion and refinement.

## 3 Results

In this section, we document learnings from the evaluation, drawing initially on the project records to chart the process of establishing the Advisory Group and involvement activities (Phase 1). Members' views on the quality of their involvement, based on analysis of the data from both surveys (Phase 2), and the virtual World Café (Phase 3), are then presented.

### 3.1 Phase 1: Insights From Project Records

#### 3.1.1 Establishing the Advisory Group

In March 2020, the RE:CURRENT Principal Investigator (K.O.D.) wrote to knowledge users involved in the provision, governance, management or receipt of recurrent miscarriage services and supports, across the Republic of Ireland, inviting them to join a Research Advisory Group for a national evaluation of recurrent miscarriage care. Potential members were identified by the research team based on their professional networks and knowledge of the Irish context, aiming to ensure a variety of knowledge user types, disciplines, geographical areas and service types were represented. As it can be challenging to recruit men to reproductive and pregnancy loss research [51, 52], at their first meeting, the research team asked Advisory Group members for assistance in identifying men that might be interested in joining; two men were invited, and joined, in May 2020. Members were also asked to identify any additional knowledge users that should be invited to participate; none were suggested.

#### 3.1.2 Membership

All 23 knowledge users invited to join the Advisory Group accepted the invitation. One parent advocate resigned from the group in October 2020 due to personal circumstances, thus the Group comprised 22 members for the remainder of the project (details in Table 4), though some were absent at various stages due to leave (e.g., maternity). In consultation with the Group, we did not replace any members as we felt that we had sufficient input from their category. In addition, we did not want to alter group dynamics by bringing in new people at later stages in the project. All but one member (a parent advocate) were women.

**Table 4:** Composition of the Advisory Group.

Category	Details
Health Professionals (HP, *n* = 11)	Consultant Obstetricians and Gynaecologists (*n* = 5) Clinical Midwife/Nurse Specialists in Bereavement and Loss (*n* = 4) Clinical Nurse Specialist in Perinatal Mental Health (*n* = 1) General Practitioner (*n* = 1)
Administration, Governance and Management (AGM, *n* = 7)	People involved in the administration, governance and management of maternity services including a representative from the fertility sector
Parent Advocates (PA, *n* = 4)	Women and men with lived experience of recurrent miscarriage and/or from support and advocacy groups

*Note:* ‘Parent advocate’ is the term agreed upon by members within this category; we appreciate that people have varied preferences regarding pregnancy loss [53] and lived experience [54] terminology.

We did not seek for any members, particularly PPI contributors, to ‘represent’ the perspectives of everyone with a particular type of experience. People can play multiple roles within a project (e.g. based on their lived experience, job/occupation and/or as a tax payer) [55] and we recognised the need for a diversity of roles, expertise and experience [56, 57]. We facilitated this to some extent by involving four different PPI contributors, each of whom brought different perspectives; two had prior involvement with the research group.

#### 3.1.3 Terms of Reference

Draft terms of reference for the Advisory Group were prepared by the research team based on the grant application which had knowledge user/PPI input. These specified that the Group would provide expert and experiential advice on the design, conduct, interpretation and dissemination of the research. They outlined membership and roles and responsibilities of the research team and the Advisory Group, as well as governance and administrative structures. Clear role descriptions and responsibilities for PPI contributors are important in facilitating effective partnerships [42]. The terms of reference were discussed at the first meeting of the Group, and subsequently sent to members for feedback and formal agreement. This was a ‘live’ document and evolved as the research developed. Terms were updated in September 2020, with Group approval, to specify the criteria for authorship of research publications and the protocol for dissemination of research at academic/professional events.

The term of the Advisory Group initially was to run from January 2020 to December 2021, with six meetings proposed for this period. However, the project was extended to July 2022 on receipt of funding to facilitate the meeting of project deliverables following COVID‐19 interruptions. All members agreed to the extension of the Group's term and three additional meetings were held, nine meetings in total.

#### 3.1.4 Meetings

The first meeting of the Advisory Group was planned for April 2020. However, in March 2020 COVID‐19 was declared a global pandemic by the World Health Organisation, with resultant social lockdowns ordered in the Republic of Ireland. Therefore, the research team proposed a virtual meeting to initiate contact. With ongoing restrictions, the virtual meetings continued for the duration of the project (see Supporting Information S1: File 3). Initially conducted via Zoom, later meetings were held through Microsoft Teams due to changes in institutional policies. Doodle (a free online meeting scheduling tool) polls were used to establish meeting times; evenings appeared to suit the majority, and this was maintained throughout. Agendas were circulated in advance of meetings, along with supporting documentation. Each meeting had an assigned Chairperson. Meetings focused on the presentation and discussion of research plans, progress and findings, with space and time for Advisory Group input. They also provided an opportunity for members to express interest in being more involved in particular studies or activities outside of formal group meetings (see Table 1); follow‐up was then initiated by member(s) of the research team. Minutes taken by the research team during meetings were circulated for review.

#### 3.1.5 Communication

Beyond research meetings, members were in regular contact with the research team (and vice versa) and were encouraged to engage with questions, feedback and/or ideas to facilitate their involvement. Members provided written/verbal feedback on electronic/printed documents (depending on their preferred modes) and were also in contact via telephone/video call, as needed, during research activities. Throughout, the research team reiterated the voluntary nature of involvement activities, that members could engage—or not—as they sought fit, and that their time and expertise were valued.

At the first meeting, parent advocates suggested that it would be beneficial to increase public awareness of the work being done on the RE:CURRENT Project. The research team published regular project updates [31]—providing information and updates to Advisory Group Members and other knowledge users—between meetings to keep people informed, engaged and involved in research and knowledge translation activities. Seven members of the Advisory Group also contributed to these updates, providing statements about their involvement experiences (Supporting Information S1: File 4). The inclusive nature of the Group, the learning acquired, and the potential to impact services and supports were frequently mentioned. Members also helped to disseminate project updates via their networks.

#### 3.1.6 Involvement in Research Activities

The Advisory Group engaged in various research activities across the project and within the individual studies from conception and throughout (see Table 1). Members also contributed to aspects of the research in small groups, or individually, depending on the study requirements and their area of expertise.

#### 3.1.7 Short‐Term Outcomes of Involvement

Project records—including researcher diaries, meeting minutes and project updates—highlighted the benefits of different perspectives and expertise on research processes and outcomes. As researchers with varying levels of expertise, we gained a better understanding of the area and an increased awareness of knowledge user issues. Knowledge users reported gaining practical skills, knowledge about research and topics, and a peer support network; in addition, parent advocates reported increasing confidence as advocates and a sense of empowerment.

Arising from RE:CURRENT, we successfully secured a Knowledge Translation Award from the Health Research Board to enhance the translation of the RE:CURRENT Project learnings into policy and practice. Some Advisory Group members were involved in this award which ran from December 2022 to November 2023 and produced a series of policy briefs, case studies, information booklets, multi‐lingual videos and other resources [31]. Some were also involved in writing/reviewing a national clinical guideline for recurrent miscarriage and associated clinical and plain language summaries [58].

### 3.2 Phase 2: Insights From Involvement Quality Survey

The survey aimed to enable members to share their views and experiences of involvement in the Project as the project progressed. The size of the Advisory Group was large in terms of an involvement endeavour, however, small in terms of being able to draw firm conclusions from the data. We therefore present the data as a means of highlighting members' feedback, rather than any statistical changes in views or experiences between the two timepoints.

Seventeen out of 21 Advisory Group members completed the first round of the survey in February 2021 (T1; 81% response rate). They self‐identified as consultants (*n* = 4, 23%), midwives (*n* = 4, 23%), other health professionals (*n* = 3, 18%), governance and management (*n* = 2, 12%), support group representatives (*n* = 1, 6%), parent advocates (*n* = 2, 12%) and other (*n* = 1, 6%). Twelve out of 21 Advisory Group members completed the second and final round of the survey in May 2022 (T2).

Participants' responses to both rounds are presented in Table 5. In general, categories in the ‘research context’ domain were more highly rated than those in the ‘personal factors’ domain, though the ‘sense of being’ category was rated highly in the former. Items relating to ‘ability’ and ‘potential’ rated less highly, with T1 and T2 summary scores of 14/24 (58%) and 16/24 (67%), respectively for ‘ability’, and 9/16 (56%) and 11/16 (69%) for ‘potential’. This is perhaps unsurprising as the Advisory Group was established in May 2020 when the project was underway; they were not involved in the initial project proposal/grant application process. In addition, while the project championed the involvement of knowledge users, their involvement focused on particular areas such as the generation of KPIs, and guiding the conduct and dissemination of research.

**Table 5:** Summary of participant responses from both rounds of the survey, by domain, category and item.

		Mean (range)		
		T1	T2	
		N = 17/21	N = 12/22	T2 – T1
*Personal factors*				
Ability (to):	Achieve your own goals through the research	2 (3)	3 (3)	+1 (0)
	Make a contribution to the research	3 (3)	3 (3)	0 (0)
	Make decisions about how to do the research	2 (3)	2 (2)	0 (−1)
	Express your views about research topics	3 (3)	3 (2)	0 (−1)
	Discuss research issues	3 (3)	3 (2)	0 (−1)
	Take on new research challenges	2 (4)	2 (3)	0 (−1)
	*‘Ability’ summary score*	*14/24 [58%]*	*16/24 [67%]*	*[+9%]*
Potential (to):	Choose the type of role you play in the research	2 (3)	2 (2)	0 (−1)
	Bring your own ideas and values to the research	3 (1)	3 (3)	0 (+2)
	Work in ways that suit you	2 (3)	3 (3)	+1 (0)
	Gain status, expertise or credibility because of your involvement	2 (4)	3 (3)	+1 (−1)
	*‘Potential’ summary score*	*9/16 [56%]*	*11/16 [69%]*	*[+13%]*
Sense of being	Valued as a partner (not controlled)	3 (4)	4 (3)	+1 (−1)
	Enabled (rather than constrained)	3 (3)	3 (3)	0 (0)
	Empowered (rather than exploited)	3 (3)	3 (3)	0 (0)
	Consenting (happy to be involved) not coerced (unhappy about it)	3 (2)	4 (1)	+1 (−1)
	It is accepted that different people have different responsibilities and decisions to make about the research	3 (2)	4 (1)	+1 (−1)
	*‘Sense of being’ summary score*	*16/20 [80%]*	*18/20 [90%]*	*[+10%]*
*Research contexts*				
Research relationships	The researchers have the right reasons for wanting to work with you	3 (2)	4 (2)	+1 (0)
	There is sufficient funding to make involvement work	3 (4)	3 (3)	0 (−1)
	You have enough information about involvement	3 (2)	4 (2)	+1 (0)
	The way the researchers work with you is supportive	3 (2)	4 (2)	+1 (0)
	The way the researchers communicate with you is supportive	4 (2)	4 (2)	0 (0)
	The types of goals that the researchers want are what you want	3 (2)	4 (2)	+1 (0)
	*‘Research relationships’ summary score*	*19/24 [79%]*	*21/24 [88%]*	*[+9%]*
Ways of doing research	There is a clear role in the research for you	3 (3)	3 (3)	0 (0)
	The skills/experience needed for the role are clear to you	3 (3)	3 (3)	0 (0)
	The responsibilities for the role are clear to you	3 (3)	3 (3)	0 (0)
	You are aware of the legal and ethical ‘rules’ for doing research (e.g. confidentiality)	3 (2)	4 (1)	+1 (−1)
	*‘Ways of doing research’ summary score*	*11/16 [69%]*	*13/16 [81%]*	*[+12%]*
Research structures	Noticed and recorded as part of the work of the organization	3 (3)	3 (3)	0 (0)
	*‘Research structures’ summary score*	*3/4 [75%]*	*3/4 [75%]*	*[0]*
Overall individual score		73/104 (51) [70%]	82/104 (58) [79%]	+9 (+7) [+9%]

*Note:* Participants rated their responses to each question from 0 to 4, where 0 is ‘not at all', 1 is low and 4 is high; some summary scores may not total from individual scores due to rounding.

### 3.3 Phase 3: Insights From Virtual World Café

During the World Café, members of the Advisory Group and research team reflected on what worked well regarding involvement in the project, what could be done better, and recommendations for the future. We generated two themes during the analysis of this data: structural space, and relational space. Each of these, with accompanying sub‐themes, is discussed below; illustrative quotes are available in Supporting Information 1: File 5.

#### 3.3.1 Theme 1: Structural Space

This theme captures the everyday and higher‐order structures that shape research and involvement activities including research administration, research protocols and the policies and practices of academic institutions and publishers.

##### 3.3.1.1 Meeting Accessibility

The time and location/format of meetings impacted accessibility for Advisory Group members and their involvement experiences. They stated that the notice period for meetings was sufficient and meetings were well organised and ‘methodical’ but there were ‘divided opinions’ on scheduling. Parent Advocates were satisfied with evening meetings indicating that they accommodated work and family life; however, other members preferred meetings held during the working day.

Advisory Group and research team members believed that, although not part of the original protocol and dictated by COVID‐19 restrictions, ‘the use of virtual means’ to hold meetings worked well. Online meetings facilitated attendance allowing members across the country to attend consistently, which might not have been possible otherwise. They noted, however, that in‐person meetings might have been useful in building rapport and clarifying information. Acknowledging the benefits of both formats, it was agreed that a ‘hybrid approach’ would be ideal to increase accessibility.

##### 3.3.1.2 ‘Peeling the Onion’ Takes Time and Space

While Advisory Group members spoke positively about the ‘comprehensive and lateral thinking' of the research team in ‘peeling the onion for every opportunity to make this project worthwhile' in terms of research activity and involvement, they also highlighted challenges with the time commitment required and the lack of clarity at the outset around this. The research team discussed how they experienced the project as an evolving learning process and that while there was a structured plan for the research, some of the tasks—such as KPI development—took longer than expected. They felt that they had been ambitious in what they wanted to achieve and that a more ‘realistic protocol’ would have been beneficial. Additionally, the team acknowledged that the protocol had not factored in sufficient time for the involvement that was facilitated throughout the project.

##### 3.3.1.3 Operational Obstacles

The research team noted that while PPI is now expected to be an inherent part of research, institutional and publication policies challenged involvement efforts. They highlighted limited funding within the project to support the desired level of involvement and how institutional policy restricted their autonomy in how members were reimbursed (parent advocates received a voucher for each meeting, a nominal amount; other knowledge users participated as part of their professional roles/jobs).

They also noted that academic publishing processes created obstacles in the inclusion of members as co‐authors, for example, requiring them to complete complicated online forms to be named.

#### 3.3.2 Theme 2: Relational Space

This theme captures the social space in which involvement took place and it highlights how interpersonal relationships shaped the nature and experience of involvement activities.

##### 3.3.2.1 Different Backgrounds at the Table

Advisory Group and research team members spoke positively about the multidisciplinary composition of the Advisory Group. However, they felt that due to the size of the Group, it was not fully clear who was involved at all times. Reflecting on who else could have been invited, they suggested additional representation from primary care and potential funding bodies with a view to increasing support for the implementation of project recommendations.

##### 3.3.2.2 Supportive Relationships

Advisory Group members described a welcoming and ‘collegial’ atmosphere within the project that was considerate of the difficult subject matter at hand and in which they felt they were given space to contribute without pressure or expectation. They discussed how this fostered open communication, making it easy to ask questions and to give feedback during meetings and in the conduct of research activities. They valued the ‘efficiency and responsiveness’ of the research team and the supportive environment created. Parent Advocates in particular expressed appreciation for the accessibility of the research team and the opportunity to reach out for support outside of normal working hours and through various communication channels.

Emphasising that the commitment of the Advisory Group extended beyond the large group meetings, and that knowledge user groups and individuals were called on at different times to contribute to various tasks, the research team admitted to feeling a heavy sense of responsibility about their facilitation of involvement activities. They described worrying about the nature and volume of work they were asking from members, and the potential burden of involvement activity. They described a sense of ‘comfort’ in the open and honest lines of communication that were utilised by members to voice issues when needed.

##### 3.3.2.3 Dependent on Clinical Expertise

The KPI development process stood out from other aspects of the project as a particularly difficult endeavour for members. The Health Professionals group expressed concern that the process might have been challenging for Parent Advocates. This was echoed by the Parent Advocate Group who reported feeling ‘uncertain and daunted’ by the Delphi Study. They highlighted the weight of responsibility they felt in representing the ‘parents voices/experience’ in this exercise and voting on items that they didn't fully understand but that they believed would impact people's lives. The Governance and Management group stated that they learned a lot about recurrent miscarriage from the process, but some found the Delphi Study ‘overwhelming’. While members appreciated lay explanations provided by clinical colleagues, they felt dependent on others’ clinical expertise to inform decision‐making. Advisory Group and research team members felt that smaller preparatory meetings between the research team and non‐clinical members in advance of the larger Delphi study meetings would have been beneficial in ‘creating a leveller playing field for meaningful participation'.

## 4 Discussion

In this section, we reflect on findings from the evaluation of involvement activity within a national evaluation of recurrent miscarriage services. We combine insights from the three components of the evaluation—project records, surveys and World Café, under two central themes: the relational nature of knowledge user involvement and supportive structures, and end with a brief discussion around outcomes and impacts. Key lessons learned are summarised in Box 1. Our findings reinforce observations that PPI and research co‐production are profoundly relational and interactional processes [8, 30]. While our findings largely align with existing PPI literature, they provide further insights into how to involve and support a broad range of knowledge users across a project as part of integrated knowledge translation efforts. As PPI frameworks and toolkits are being produced on ‘an industrial scale’ [59], more structural support is needed to support, sustain and maximise opportunities within research coproduction.

BOX 1 **Supporting meaningful research coproduction: Key lessons learned**

**Table:** 

Have shared values and goals, while embracing diversity As a research team—have shared values around research coproduction (including the value of different types of knowledge) and a shared vision of how it can be implemented within your project. Build a shared vision or goal for the research project and what you hope to achieve (e.g. policy and/or practice impacts) amongst all involved. Acknowledge, value and support a diversity of knowledge user (including PPI) roles, expertise and experience. Implement—and advocate for—supportive structures Recognise and advocate for the institutional and financial supports needed to facilitate meaningful involvement, for example, adequate funding for reimbursement and institutional policies to enable this in a way that works for knowledge users. Be aware of structural challenges to involving knowledge users in academic publication processes and support their inclusion and involvement in these. Appreciate that knowledge translation activities can extend way beyond a funded project's timeline and plan for ways of managing this, including expectations of all involved, and how knowledge users can be involved and supported, should they wish to be, beyond the project end date. Consider a long‐term programme—rather than project—approach to research co‐production. Plan for involvement Set realistic study protocols that allow time and space for the evolving nature of research with knowledge users, and be clear about the time commitment involved from the outset. Have clear terms of reference, agreed with knowledge users, to guide the work, and actively review them. Appreciate that knowledge users—particularly people with lived experience—may question their ability and knowledge to meaningfully participate in research coproduction and may feel a ‘weight of responsibility’ in ‘representing’ lived experience perspectives. In addition to implementing other learnings outlined, being clear from the outset of the importance and scope of their contributions is important, as well as working with knowledge users to identify ways to support their involvement (e.g., build in time to develop information materials about research terms and processes, facilitate training and development opportunities). Consider having a forum for PPI contributors to meet in advance of larger group meetings to enhance their involvement. Have people with the necessary knowledge and skills within your team to support knowledge user involvement. Have an experienced facilitator lead meetings to create a supportive environment and level power imbalances. Build relationships Prioritise the relational nature of involvement over instrumental aspects or techniques Don't make assumptions about what people know or not, regardless of whether they have professional or lived experience or not. Involve knowledge users in discussions and decisions from the outset (ideally at idea generation/grant application stage)—to distribute power, build sense of ownership and generate support. Acknowledge the voluntary nature of involvement activities and that people's time and expertise are valued. Provide regular project updates to keep people informed, engaged and involved in research and knowledge translation activities; include updates on what changes have occurred based on knowledge user feedback. Foster tailored, flexible and open communications Use tailored or flexible approaches to involvement, recognising that knowledge users' needs and desired levels of involvement will vary, as will the times and ways within which they can engage in activities. Have open communication processes; encourage people to engage with questions, feedback and/or ideas to facilitate their involvement as they see fit. Provide information in accessible ways, for example, avoid jargon and use plain language. Have options to participate in activities in‐person, remotely and/or in hybrid ways to facilitate inclusion; having some in‐person (with hybrid option) meetings may foster greater rapport building. Be open and honest about challenges encountered. Create space for critical discussion and changing direction when needed—in the research itself and/or in involvement activities.

### 4.1 The Relational Nature of Knowledge User Involvement

Good relationships, time, flexibility and responsivity facilitated involvement in our project [42]. Successfully embedding involvement is dependent on the experiences and attitudes of researchers who can find PPI both rewarding and challenging [17]. As researchers, we experienced ‘personal challenges’ [3], questioning power dynamics and what we were asking of Advisory Group members given our constrained resources to support research coproduction. It is evident from the survey and World Café data that Advisory Group members appreciated these challenges and the honesty and support from the research team, as observed by others [30]. Our insights demonstrate that positioning researchers as the group holding power within research coproduction can mask the complexity of power relations; for example, researcher power may be constrained by institutional or funder processes and structures, or their employment status [17, 60]. Researchers need to recognise the limits of their roles and capacity in addressing structural issues in affecting change, and be prepared for the moral distress that can arise [61].

Building and sustaining trusting relationships with PPI contributors and ensuring they are supported can involve a balancing act between work and private lives [23, 39]. This applies to all parties involved in our experience, and supporting meaningful research coproduction requires flexibility on the part of researchers and associated institutional supports. Power imbalances, and use of jargon, are commonly reported barriers to patient involvement [42, 62]. This was observed in comments surrounding the KPI development process, where there was a reliance on clinical expertise. It is possible however that the combination of expertise enabled deeper insights into the issues under consideration.

### 4.2 Supportive Structures

In both surveys, members rated the overall quality of their involvement highly; however, areas related to potential and ability scored lower. As previously mentioned, this is perhaps to be expected as they were not involved in the initial project proposal and their involvement was somewhat constrained. Funding systems limit the extent to which researchers can share power with knowledge users as they do not allow for meaningful research coproduction during grant planning stages [63]. While the involvement of knowledge users, especially PPI, can be an application requirement, funding timelines and expected deliverables often do not allow for meaningful or sustained involvement [64]. In addition, time spent on relationship building and engagement is not always acknowledged, or supported, by universities [65]. While involvement costs were included within our project, they were under‐estimated. Reimbursement may not be a motivator of involvement for bereaved parents [66]; however, compensation for out‐of‐pocket expenses, time and expertise is important in valuing and facilitating PPI [25, 30, 42, 67]. Due to institutional requirements, we used vouchers which are not ideal [68]. Acknowledging people's contributions through co‐authorship is a potential way of legitimising and valuing alternative forms of knowledge [25]; while we tried to incorporate as much as possible, structural barriers were experienced. Greater organisational/institutional supports for knowledge user involvement, particularly PPI, are needed [17].

The right environment for participation is needed [68]. Similar to others [69], we found that the virtual environment had a ‘democratising’ effect on the meetings. As noted during the World Café, it also afforded opportunities for increased participation, which may have been more difficult to achieve with in‐person meetings. That said, Advisory Group members felt that some in‐person or hybrid meetings would have been beneficial; benefits of hybrid meetings may depend on the activity or area of focus [68]. In addition, members valued the skilled facilitation of meetings, which can help to create supportive environments and level power imbalances [62], and enhance the quality of online involvement activities [69]. The need for PPI contributors to have a forum on their own in advance of broader knowledge user meetings was highlighted in our evaluation, and in other studies also; the funding and time implications associated with this need to be considered [23].

Challenges with the time commitment required and the lack of clarity at the outset were highlighted during World Café discussions. Grant funding is often time limited, and is a barrier to sustainable involvement [70]. This can be compounded by the fact that many involvement activities are carried out by early career researchers who can be on short‐term contracts [17]. The RE:CURRENT project officially ended in July 2022 (with additional support until April 2023 to finalise publications); however, at the time of writing, dissemination activities continue and some members of the Advisory Group remain involved. As such, there is no ‘end’ to the process of knowledge translation—but rather, the ripples of influence continue beyond the study timeframe [38]. A long‐term programme—rather than project—approach to research coproduction is needed [71].

### 4.3 Outcome/Impacts of Research Coproduction With Knowledge Users (With Embedded PPI) as Part of a Knowledge Translation Strategy

Several outcomes of knowledge user involvement are reported, such as increased efficiency, quality and relevance of research and outputs; mutual learning and skills acquisition; new projects; realisation of personal/professional goals, feeling valued, increased confidence, extended social and support network and/or increased chances on future employment; awareness of research and use of research in decision‐making [3, 72]. While we did not explicitly focus on outcomes/impacts in our evaluation, many of the aforementioned outcomes were observed in Advisory Group members' reflections and contributions, and by the research team. Engaging people with lived experience and other knowledge users helped to remind us of what was important in our work, enriched the process, and sustained our commitment to improving services [17, 73]. As noted elsewhere, people with lived experience reported several personal benefits to their involvement in the project, such as gaining skills, knowledge, confidence and a peer support network [3, 42]; as did other knowledge users [3]. We realised several short‐term outcomes outlined in our logic model; knowledge translation activities continue to realise longer‐term outcomes.

### 4.4 Strengths and Limitations

Our evaluation would have benefitted from individual interviews with all involved to explore perspectives that people may be reluctant to voice in the group setting, and/or with the research team; however, we were limited by available resources. An independent evaluation could have revealed insights which might not have been accessible to the research team. However, it is not uncommon for involvement activities to be evaluated by the research team and knowledge users directly involved [42]. We did not quantify time spent on involvement activities, nor did we formally measure outcomes or impacts. Given our limited resources and timelines, we focused our efforts on building and sustaining relationships to realise involvement within the Project and to deliver on its work packages. We prioritised diversity in knowledge user types, disciplines, geographical areas and service types when recruiting members of the Research Advisory Group given the broad focus of the project; future work should incorporate measures of diversity and inclusion in group composition.

## 5 Conclusions

Involving knowledge users, including PPI, within a large research project as part of an integrated knowledge translation approach is feasible, and there are opportunities to maximise the experience for all. Research Advisory Group members reported a positive and rewarding experience with visible impact on the research process. They highlighted issues with the feasibility and scope of the research protocol and challenges to autonomous involvement in aspects of the research which they felt relied on clinical expertise. Our learning reinforces that the relational nature of involvement takes precedence over instrumental aspects/involvement techniques. Realistic research protocols that allow time and space for the evolving, relational nature of research with knowledge users, and institutional and financial support to facilitate meaningful involvement—including adequate resourcing and reimbursement, are needed.

## Author Contributions

**Marita Hennessy:** conceptualisation, data curation, formal analysis, investigation, methodology, project administration, writing–original draft preparation, writing–review and editing. **Rebecca Dennehy:** conceptualisation, data curation, formal analysis, investigation, methodology, project administration, writing–original draft preparation, writing–review and editing. **Hannah O'Leary:** writing–review and editing. **Keelin O'Donoghue:** conceptualisation, funding acquisition, methodology, supervision, writing–review and editing. **RE:CURRENT Research Advisory Group:** methodology, writing–review and editing.

## RE:CURRENT Research Advisory Group Members

Una Cahill, Ríona Cotter, Mairie Cregan, Carrie Dillon, Dr Linda Drummond, Angela Dunne, Dr Minna Geisler, Dr Trish Horgan, Dr Azy Khalid, Con Lucey, Mary McAuliffe, Dr Moya McMenamin, Dr Yvonne O'Brien, Orla O'Connell, Anne O'Flynn, Aideen Quigley, Margaret Quigley, Rachel Rice, Professor Noirin Russell, Jennifer Uí Dhubhgain, Anna Maria Verling and Jill Whelan.

## Ethics Statement

Ethical approval for the study—‘RE:CURRENT Involvement Evaluation’—was granted by the Clinical Research Ethics Committee of the Cork Teaching Hospitals (CREC) [Reference number: ECM 4 (gg) 10/11/2020].

## Consent

Written informed consent for publication of the Research Advisory Group members' details was obtained from each member.

## Conflicts of Interest

The authors declare no conflicts of interest.

## Supporting information

Supporting information.

## Data Availability

The data that support the findings of this study are available on reasonable request from the corresponding author. The data are not publicly available due to privacy or ethical restrictions. A request is considered reasonable where the intended use for the data is clearly outlined, and where this intended use does not violate the protection of participants, or present any other valid ethical, privacy, or security concerns.
