# Correction to “Involving Knowledge Users in Health Services Research: Collective Reflections and Learning From a National Evaluation of Recurrent Miscarriage Services”

**DOI:** 10.1111/hex.70525

**Published:** 2025-12-05

Hennessy, M., Dennehy, R., O'Leary, H., O'Donoghue, K., and RE:CURRENT Research Advisory Group (2024), “Involving Knowledge Users in Health Services Research: Collective Reflections and Learning From a National Evaluation of Recurrent Miscarriage Services,” *Health Expectations,* 27: e70125. https://doi.org/10.1111/hex.70125.

Some errors were identified in Table 1 following publication of the article. The text regarding knowledge user involvement in work packages 3, 4 and 5 is incorrect, reading as follows for each in the original published article:

- Nine meetings of the Advisory Group; involvement in other work packages highlighted above.
- Participated in evaluation of quality of involvement (two surveys, World Café).
- Participated in the development of national clinical guidelines for recurrent miscarriage.
- Contributed to research papers, videos, infographics, media articles, blogs and project updates.

**Table 1:** Project activities and knowledge user involvement.

WP	Project activities	Knowledge user involvement
1	Identification, synthesis and appraisal of clinical practice guidelines for recurrent miscarriage care	Involved in development of protocol for the systematic review and the interpretation of findings. Authorship on both published papers—protocol and review (R.R.).
2	Evaluation of service provision in the Republic of Ireland against guideline‐based key performance indicators (KPIs) for recurrent miscarriage care	Agreed and participated in six‐stage process to develop guideline‐based KPIs, including two‐round modified e‐Delphi survey, four virtual consensus meetings, a final survey and virtual meeting. Authorship on published papers–development of KPIs (R.R.); service evaluation (R.C., and O.O.C.).
3	National care experience survey	Involved in devising/selecting survey questions, piloting the survey; assisted with participant recruitment (including photographs and quotes on survey flyers). Authorship on published paper (C.L. and J.U.D.).
4	Qualitative interview study of knowledge user views and experiences of recurrent miscarriage services	Informed the research questions, topic guide and study processes; assisted with participant recruitment; involved in the analysis and interpretation of findings. Authorship on three papers (to date; two published)—definition of recurrent miscarriage (C.L. and J.U.D.); impact of the COVID‐19 pandemic on care provision and experiences (C.L. and J.U.D.); service improvements (C.L. and J.U.D.).
5	Health economic analysis: (1) costs associated with the implementation of a best practice model of care; (2) impacts of receiving care	Part (2) involved analysis of survey data from work package 3. As such, Advisory Group members involved, informed the research question and study processes; assisted with participant recruitment; involved in the analysis and interpretation of findings.
6	Integrated knowledge translation	Nine meetings of the Advisory Group; involvement in other work packages highlighted above. Participated in evaluation of the quality of involvement (two surveys, World Café). Participated in the development of national clinical guidelines for recurrent miscarriage. Contributed to research papers, videos, infographics, media articles, blogs, project updates.

*Note:* R.R., J.U.D. and C.L.—parent advocates; R.C. and O.O.C.—health professionals.

Abbreviation: WP, work package.

Please refer to the corrected text in the revised Table 1 below.

We apologise for these errors.

