# Supplementary material for: Involving Knowledge Users in Health Services Research: Collective Reflections and Learning From a National Evaluation of Recurrent Miscarriage Services
Source: Health Expect. 2024 Dec 16;27(6):e70125. doi: 10.1111/hex.70125 (PMC11649583; doi:10.1111/hex.70125)
Supplement: Supplementary file 1 — Supporting information. [file HEX-27-e70125-s001.docx]

**Involving knowledge users in health services research: Collective reflections and learning from a national evaluation of recurrent miscarriage services**

**Supplementary Files**

**Supplementary File 1 World Café Rounds – Topics and Discussion Points**

| **Round 1** | **Round 2** | **Round 3** |
| --- | --- | --- |
| **What worked well?** | **What could be improved?** | **Recommendations** |
| **Inputs** | | - If using this process again what would you do the same / differently? - If a researcher / funder / friend / colleague was taking in part in a similar process, what advice would you give them? - In an ideal world, what would this process look like? - What is needed to achieve this? |
| - Overall approach to involvement - Group composition (relevant people present, anyone missing) - Researcher / Member roles within group & within project as a whole - Terms of reference - Meeting frequency - Bi-monthly project updates - Decision-making structures - Support from research team (practical, monetary, emotional) - Employer / organisational / family support | |  |
| **Activities** | |  |
| - Meeting organisation & facilitation (virtual vs face-to-face) - Contact outside of meetings - Research activities (involvement in research design, conduct, interpretation): Recruitment; KPI development; Review of materials | |  |
| **Outcomes** | |  |
| - Researcher & member experiences - Collaborative approach to research? - Enhanced research activity and outputs? | |  |

**Supplementary File 2 Additional Notes on the World Café**

In advance of the World Café, we sent information to the Advisory Group to introduce them to the methodology and to allow them to ask any questions. This contained information on the aim of the World Café, a step-by-step guide to the process, and World Café etiquette. These were revisited at the outset of the activity.

Members of the research team attended the meeting in-person at Cork University Maternity Hospital (CUMH), i.e. their worksite, while Advisory Group members joined the meeting remotely through Microsoft Teams. Using the breakout room function on Microsoft Teams, Advisory Group members were divided into three groups based on the three overarching categories of knowledge-users (Health Professionals, Governance and Management, and Parent Advocates) and the research team formed a fourth group. Once breakout rooms were established, a link to a Padlet containing the questions for each round was shared and a nominated host within each group opened the link and shared their screen. The research team dropped into each breakout room at this point to answer any questions and/or help to resolve any issues with the set-up. To simplify the process and reduce technical complications, the questions for all three rounds were shared simultaneously on the Padlet and each group had an assigned section to be populated by their designated host. The sections/questions focused on “what worked well?”, “what could be improved?”, and “recommendations?”. Each group’s contributions were visible to all, thereby facilitating the cross-pollination of ideas and connection of diverse perspectives, a key principle of the World Café approach. One group had technical difficulties and were unable to populate the Padlet but recorded their discussion points which they sent to the research team following the meeting. Ten minutes were allocated to each question before the small groups were called back to share insights from their discussions with the large group. The complete Padlet was shared with the group and the facilitator supported the “harvest” stage of the World Café in encouraging the identification of patterns and divergences across the different discussions.

We considered having external facilitation; however, we felt that the Group had worked well in this format previously. We were also mindful of the sensitivity of topics discussed. We were cognisant of the potential impact and shift in dynamics which may occur from introducing external facilitation, as many of the Advisory Group had built a rapport with the research team and with that, an element of trust and possibility for open flow of communication.

**Supplementary File 3 Details of Research Advisory Group meetings**

| **Meeting no.** | **Date** | **Outline of issues discussed** |
| --- | --- | --- |
| 1 | 18/05/2020 | - Introductions - Project overview, including detailed discussion on issues relating to the care experience survey and qualitative interview study - Draft terms of reference outlined and discussed |
| 2 | 14/09/2020 | - Update on project activities, including individual studies - Detailed presentation and discussion of: - Proposal for developing KPIs for recurrent miscarriage care - Logic model of involvement in the project and proposal to evaluate involvement activities. Need for declaration of interests / conflict of interest policy noted |
| 3-6 | 09/12/2020; 12/01/2021; 03/02/2021; 03/03/2021 | - Consensus meetings to reach agreement on which recommendations and outcomes should be included in the suite of KPIs for recurrent miscarriage care. These meetings were chaired by an external facilitator (DD) with experience in facilitating/conducting consensus activities. The process is described in detail elsewhere (1) |
| 7 | 28/04/2021 | - Feedback from members on involvement in the KPI development process was elicited with interactive online tools utilised to facilitate feedback (1) - Procedures for the care experience survey presented and discussed - Preliminary findings from qualitative interviews presented and discussed - Findings from the first of two involvement evaluation surveys were presented to the Advisory Group with feedback welcomed on those areas identified as in need of improvement |
| 8 | 19/01/2022 | - Gap of nine months since the previous meeting acknowledged; external Chair thanked the research team for not bringing the Group together until there were concrete items to discuss. Benefit of regular project updates from the research team in the interim were highlighted - Preliminary findings from the care experience survey (2) presented and discussed - Preliminary themes from the analysis of the qualitative interview data focusing on how recurrent miscarriage is defined (3) presented and discussed - Study day on recurrent miscarriage – to be held in conjunction with the Royal College of Physicians of Ireland – discussed |
| 9^th^ and final meeting | 31/05/2022 | - World Café to evaluate involvement within the RE:CURRENT Project - Preliminary findings on the impact of receiving recurrent miscarriage (4) presented and discussed - Preliminary themes from the analysis of the qualitative interview data focusing on service improvements (5) presented and discussed - Summary of overall findings of the project focusing on the need for the evidence-based management of recurrent miscarriage, the provision of supportive care, and the optimum provision or utilisation of healthcare resources. They highlighted stakeholder support for service improvements and stressed the importance of continued advocacy efforts to encourage the standardisation of care pathways for recurrent miscarriage, dedicated resources, and patient-centred care. - Formal close of the Advisory Group. Agreed that the research team would continue to contact members with study updates and for feedback on remaining research activities |

**References**

1. Hennessy M, Linehan L, Dennehy R, Devane D, Rice R, Meaney S, et al. Developing guideline-based key performance indicators for recurrent miscarriage care: lessons from a multi-stage consensus process with a diverse stakeholder group. Research Involvement and Engagement. 2022 May 14;8(1):18.

2. Flannery C, Hennessy M, Dennehy R, Matvienko-Sikar K, Lucey C, Dhubhgain JU, et al. Factors that shape recurrent miscarriage care experiences: findings from a national survey. BMC Health Services Research. 2023 Mar 31;23(1):317.

3. Dennehy R, Hennessy M, Meaney S, Matvienko-Sikar K, O’Sullivan-Lago R, Uí Dhubhgain J, et al. How we define recurrent miscarriage matters: A qualitative exploration of the views of people with professional or lived experience. Health Expectations. 2022;25(6):2992–3004.

4. Flannery C, Burke LA, Gillespie P, Hennessy M, O’Leary H, Dennehy R, et al. Economic and health-related quality of life impacts of receiving recurrent miscarriage care in Ireland: Exploratory analysis drawing on results from a national care experience survey. Reproductive, Female and Child Health. 2024;3(3):e105.

5. Hennessy M, Dennehy R, Meaney S, Matvienko-Sikar K, O’Sullivan-Lago R, O’Donoghue K. Stakeholder perspectives on recurrent miscarriage services and improvement priorities: Qualitative findings from a national evaluation. American Journal of Obstetrics & Gynecology. 2023 Jan 1;228(1):S161–2.

**Supplementary File 4 Research Advisory Group Member Contributions to Project Updates**

***Project Update 2, October 2020***


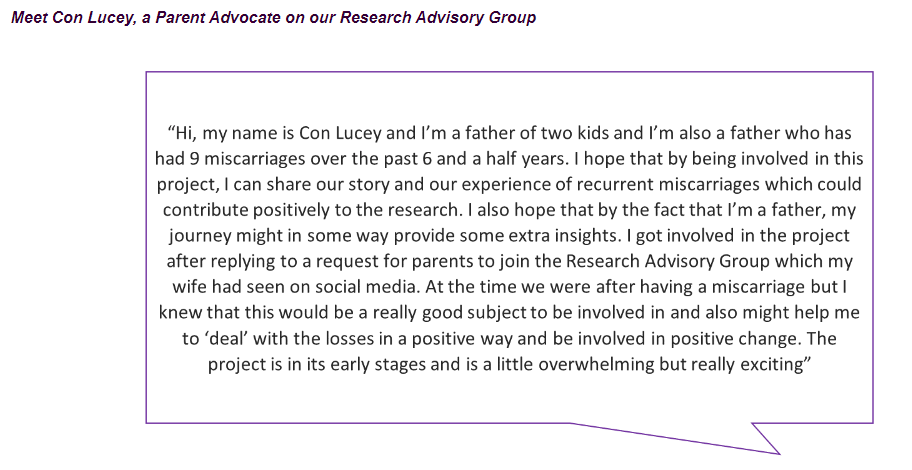


***Project Update 3, December 2020***


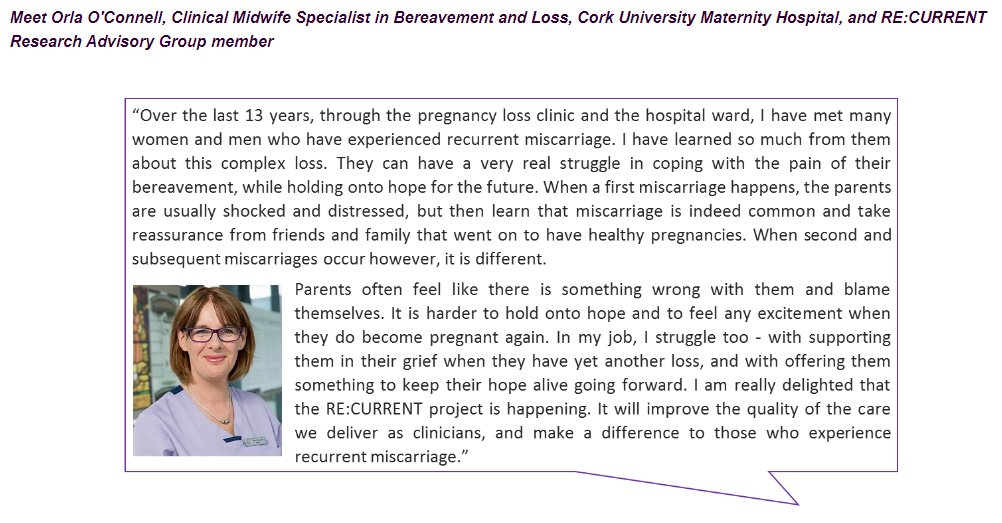


***Project Update 4, February 2021***


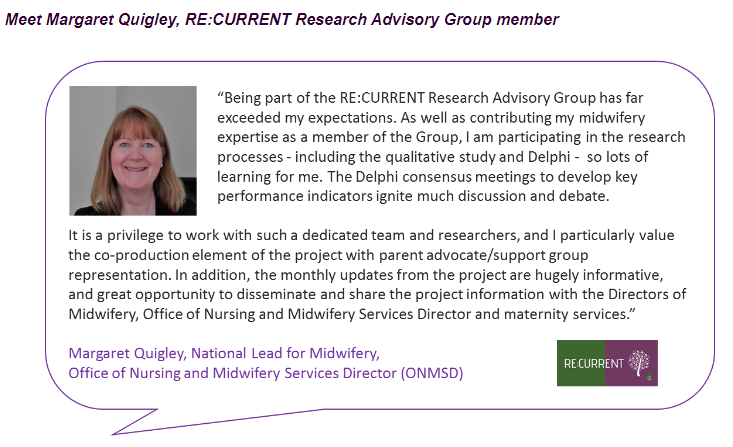


***Project Update 5, June 2021***


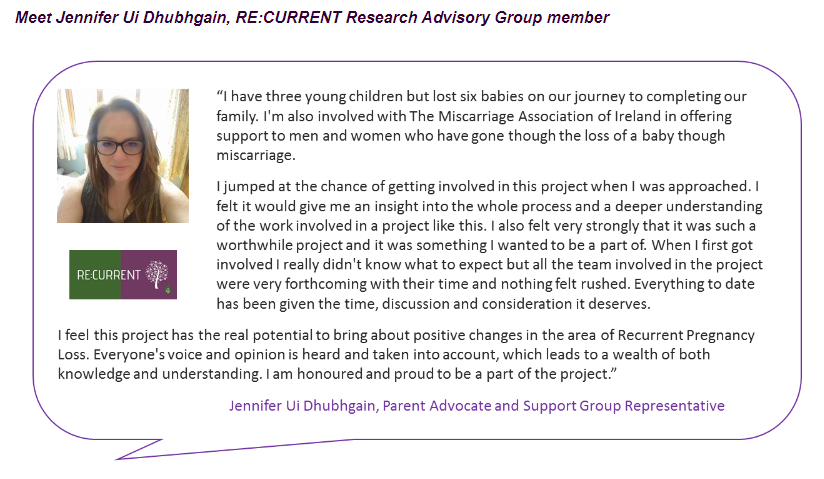


***Project Update 6, September 2021***


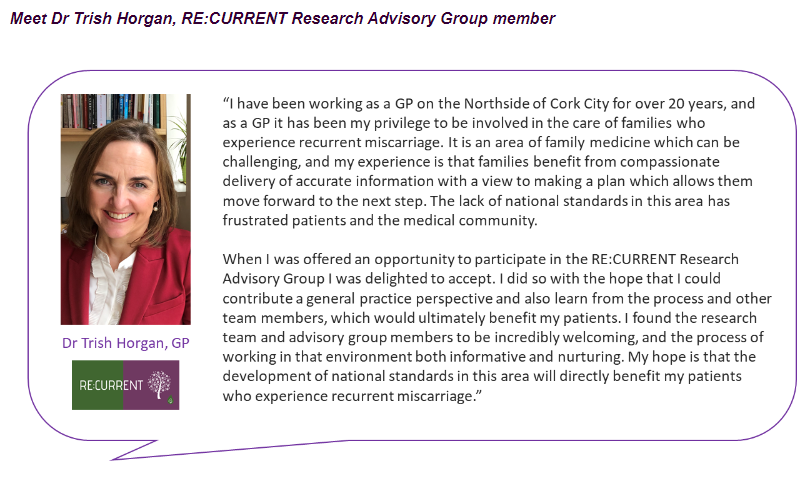


***Project Update 7, November 2021***


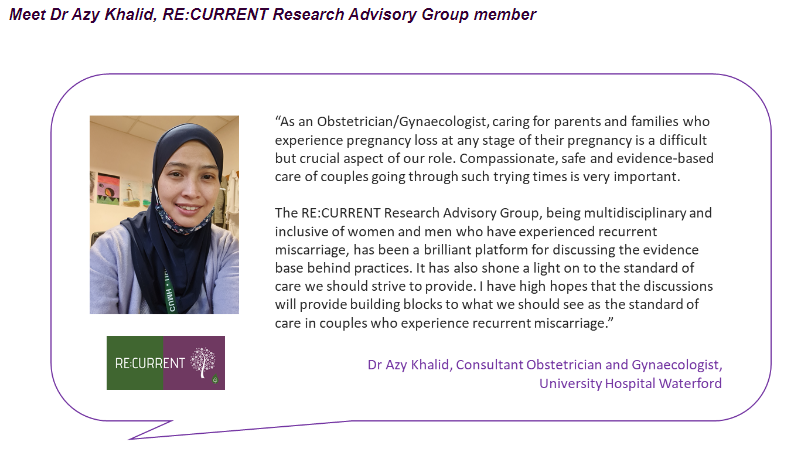


***Project Update 8, March 2022***


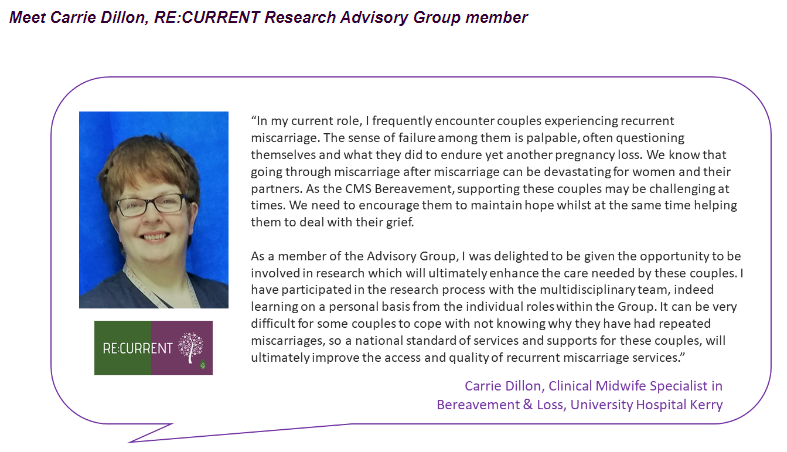


**Supplementary File 5 Overview of themes generated from the World Café data**

| **Theme** | **Selected supporting quotes** |
| --- | --- |
| ***Theme 1: Structural space*** | |
| Meeting accessibility | - “The timings of the meetings am like suited us I suppose with work and family like, you know, the kids and things like that” (Parent Advocate) - “I acknowledge that we all have different preferences for meetings. Am, you know, I felt evening meetings don't work for people. That's not the case for everybody. I would feel meetings during the working day are more preferable, I know that doesn't work for everybody and Parent Advocates said that the meetings worked for them” (Governance and Management Member) - “We couldn't come to a resolution about whether the time was good or the time was bad 4-6pm and there's kind of divided opinions on that.” (Health Professional) |
|  | - “…for the most part we had excellent attendance…I think we might have done better online than we might have done face to face ultimately in terms of attendance” (Research Team Member) - “Do you know it was good, I suppose, it meant a lot more people could attend” (Parent Advocate) - “The use of virtual means has worked very well” (Governance and Management Member) - “The technology obviously worked well for us and it allowed for kind of a broad national representation on the group which was useful” (Health Professional) |
|  | - “Consider at least one face to face meeting” (Health Professional) - “Do you know it was good I suppose, it meant a lot more people could attend. But then later on in the Padlet I would have kind of said that it would have been nice to meet face to face as well. Do you know that kind of contradicts that, but you know if I met ye like, some of you people, you know you get a lot more information if you meet someone face to face I suppose” (Parent Advocate) - “I think a mix of both, a mix of both, I think it was like the attendance was great but like I think it was me who said the face to face like I'm all about the meeting people face to face. So, you get a lot like if, it's very hard getting instructions over emails or texts or telephone calls you know, like face to face” (Parent Advocate) - “I think we said the same thing that it would have been really nice, particularly towards, well at the beginning and at the end, to meet everybody but this study began in January 2020 and you all know what happened in March 2020, so yeah, the choice got taken out of our hands initially and we definitely wouldn't, I wouldn't be designing something to be totally online. I certainly think getting people around the table, even if it's smaller groups really achieves a lot, you know” (Research Team Member) |
| “Peeling the onion” takes time and space | - “I think everybody felt that they completely underestimated the time commitment and would have maybe appreciated a better awareness of that at the outset. But then whether that would have affected participation, we don't know so there's for and against that point” (Health Professional) - “When the Advisory Group was set up, the study was already designed so the protocol was in place, but it would have been lovely to have members involved in the design stage and we might even have done better work in that instance, or maybe have done it a little differently” (Research Team Member) |
| Operational obstacles | - “Vouchers were given to parent advocates…it's what we are allowed to do within UCC” (Research Team Member) |
|  | - “When we submitted papers [co-authored with Advisory Group members] emails were then sent to all authors who were suddenly bombarded with these emails asking but what do I do here? How do I do it?” (Research Team Member). |
| ***Theme 2: Relational space*** | |
| “Different backgrounds at the table” | - “So specifically, we thought the composition of the group was representative of all those people that need to be involved. You know, you had your clinicians, you had your patient advocates, you had your research, management, governance. So that was excellent.” (Governance and Management Member) - “People really enjoyed the multidisciplinary aspect of it…and good that there was recognition of the crossover with fertility and recurrent miscarriage and seeing people who are in the fertility world being part of this as well” (Health Professional) - “Maybe this is this is partly due to the fact that there are so many people on the group sometimes you're not sure if there's representation from a certain sector in the group or not. But for example, community nursing like public health nurses and practice nurses should be if they're not already involved, should be involved. Perinatal mental health, I'm sure, are somewhere in the group, but possibly to our small group maybe were not as prominent, we weren't sure if their input and had been invited...and then finally, the question about whether somebody who's involved in funding the implementation of some of your recommendations would be invited and at a level of maybe slightly earlier than just presenting findings. Maybe you could consider having somebody involved in, even if it's just the latter stages of your research” (Health Professional) |
| Supportive relationships | - “In the Delphi the team allowed time and scope to ask questions. Even the stupid questions. So that was very welcome because that Delphi study in the beginning, as [Parent Advocates] mentioned as well you know, for those of us who are not clinical was, you know, very, very difficult really to be honest.” (Governance and Management Member) - “I sometimes had questions about parts of the project and when it popped into my head I would email straight away but wouldn’t expect a response until the next working day but every time I got a response within such a small space of time. This could have been on a late evening or weekend or a call on speaker phone while you drove home from work” (Parent Advocate) - “You send an email of a question, and they were back straight away, do you know and, I suppose like we felt supported throughout the whole thing. We felt like we could just pick up the phone and text, you know, it was, it was really good for us. Like, I've never been involved in anything like this am and, you know, always really helpful that you know that the ladies were there, almost waiting for us to ask a question” (Parent Advocate) |
|  | - “We worried constantly about meeting the needs of members and do we explain things enough? Was it clear? Did people feel overwhelmed? Were we giving people too much to do? And I suppose in that did people feel like they could say no if we asked them to do something? I think that there was that dialogue that, you know, we were able to communicate but it was something that I suppose, it's good to be honest that we did worry about that. We worried about, you know, were we giving too much at the meetings? When should we give things in advance of the meetings? And I suppose tied into that the burden of that work, like we were very conscious that you're all working, and this was in addition to your working day and your time with your families” (Research Team Member) |
| Dependent on clinical expertise | - “You know, I suppose we did feel that was it a big responsibility. Like we would have felt a very big responsibility making the vote or having to vote on these things that are going to be put into you know, KPIs” (Parent Advocate) - “Patient advocates may have found the process confusing and overwhelming” (Health Professional) - “The medical terminology was diﬃcult for non-medics/parent advocates and thus voting on KPI's left us feeling uncertain/daunted and yet we felt responsible given we were representing parents voices/experience” (Parent Advocate) - “… and then the other point which Parent Advocates also mentioned is the Delphi study, so the nonclinical members felt out of depth and it had to be led by the clinicians who had the expertise in describing the investigations procedures etcetera. And having said that, we appreciated the clarification that was given but certainly that piece, that whole clinical piece was definitely overwhelming to some degree. And I suppose, you know, we might have been unsure about what are we actually contributing here. We don't know really uh, you know, those of us who are not clinical at least don't know what we're talking about. Having said all that, I don't know how that can be addressed or improved” (Governance and Management Member) |
|  | - “We wondered about patient advocates in that when we were talking about the KPI's whether that would have been overwhelming for them and was it the best possible use of people's time…I think just leading on from that then maybe dividing it into sections. So like asking for example the patient advocacy groups to look at care pathways and so on and that might have been a better use of their time versus maybe the clinical people focusing on the in on the clinical aspects of it. And again, there's for and against splitting up groups” (Health Professional) - “We did learn a lot from the discussions in the Delphi you know. So there was lots of clinical discussions and excellent you know to learn about recurrent miscarriage. So the whole thing was a school day for us as well, yes” (Governance and Management Member). |
